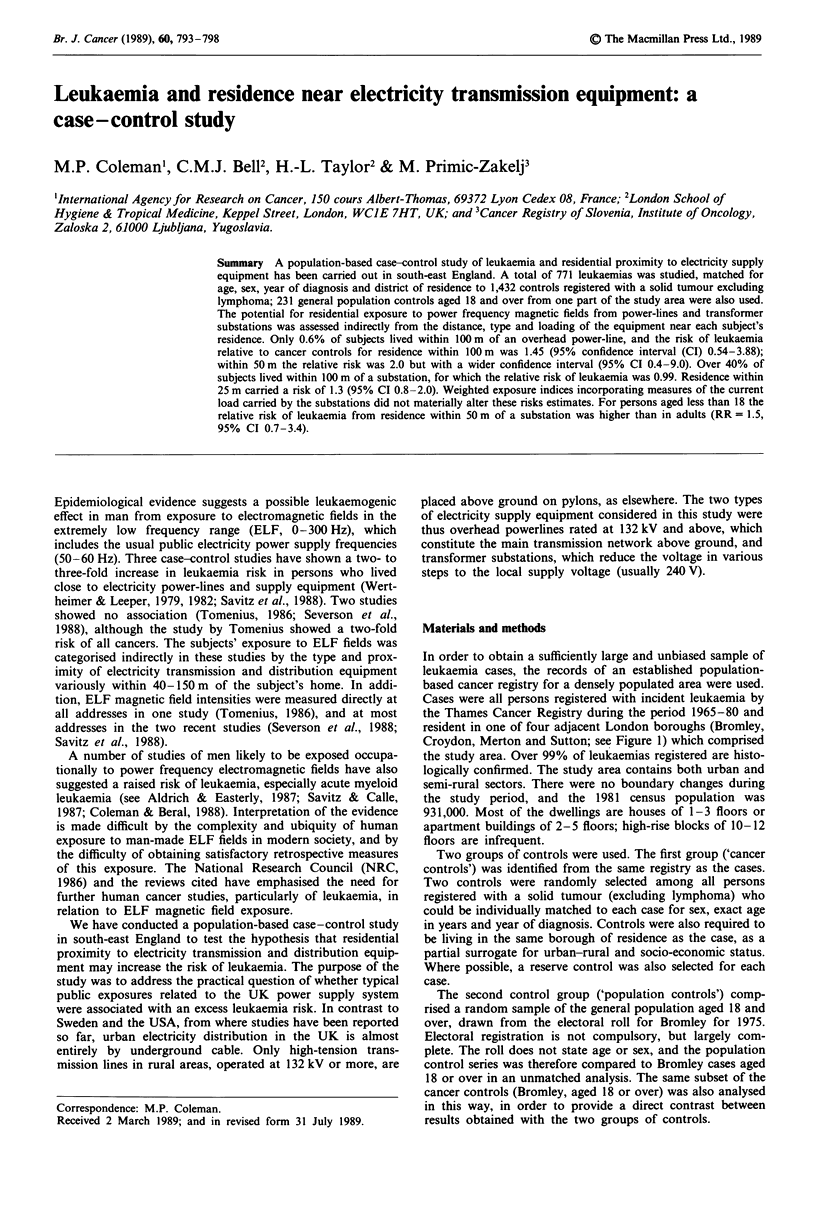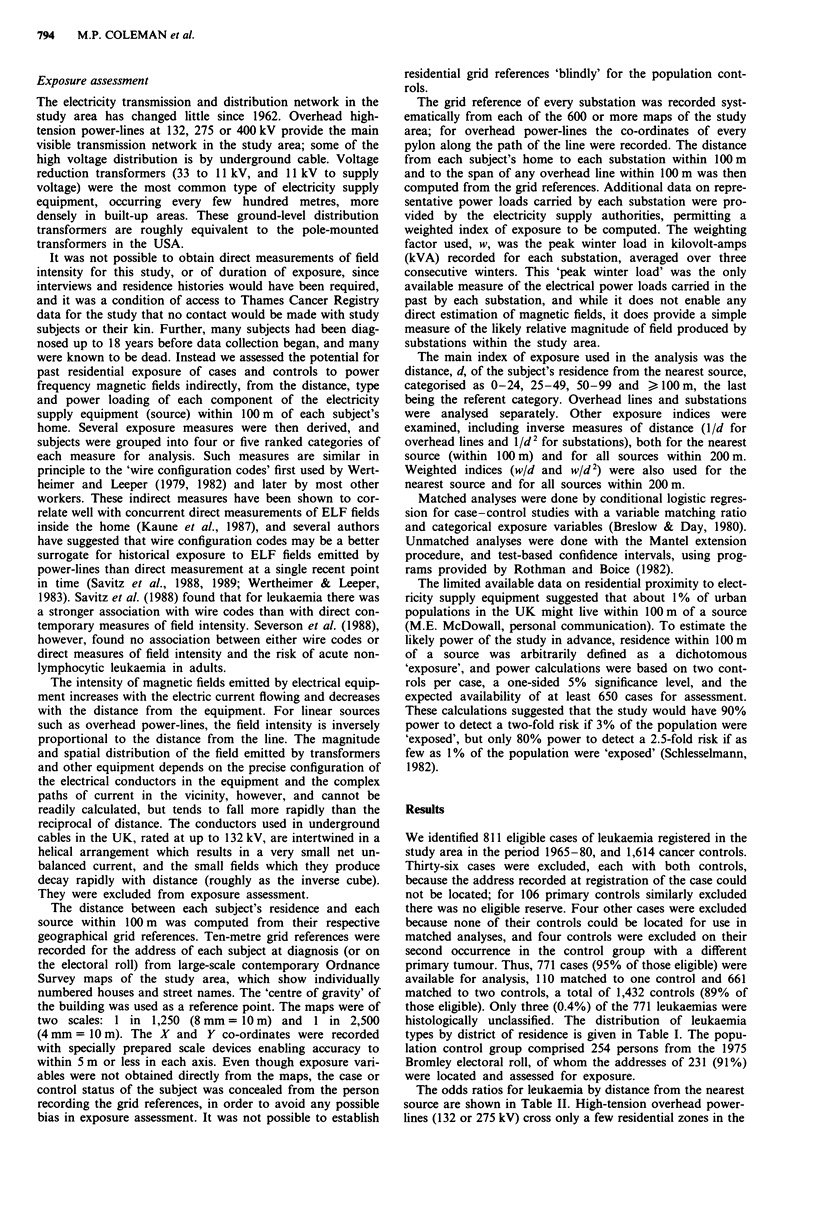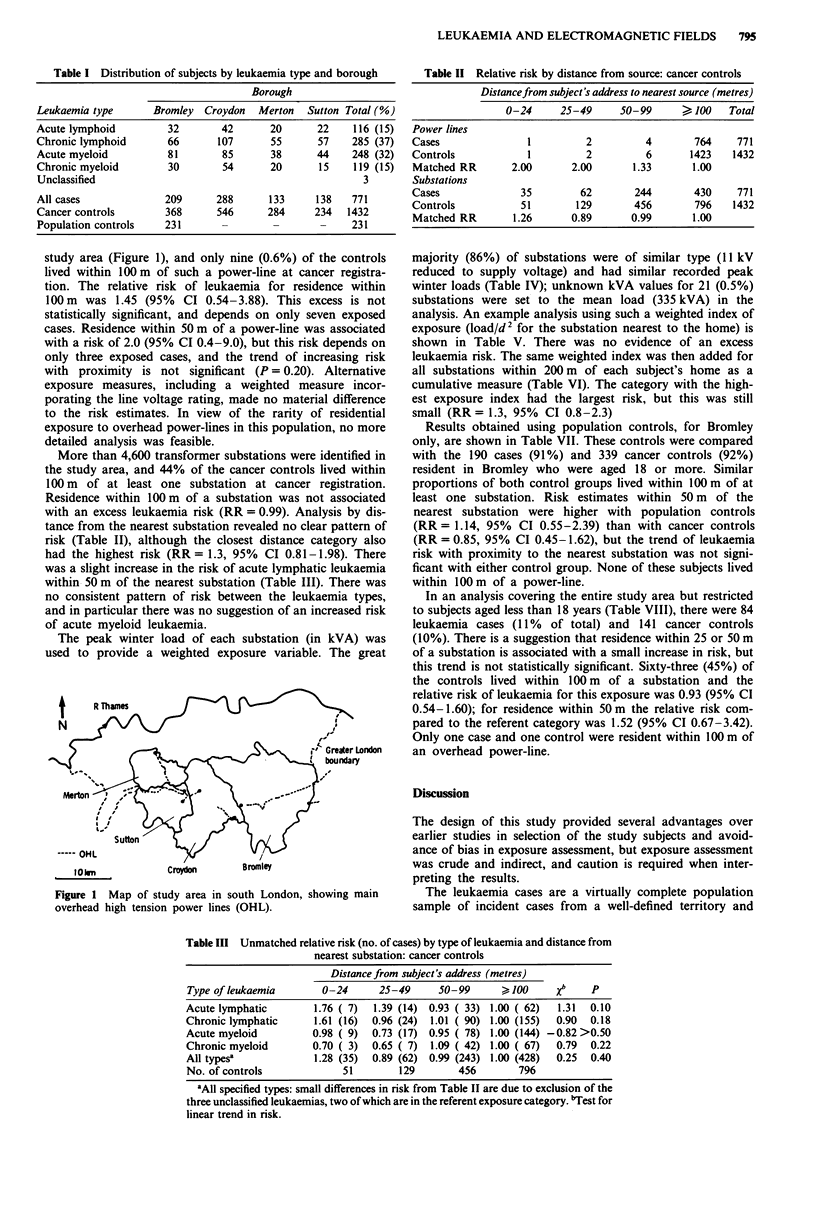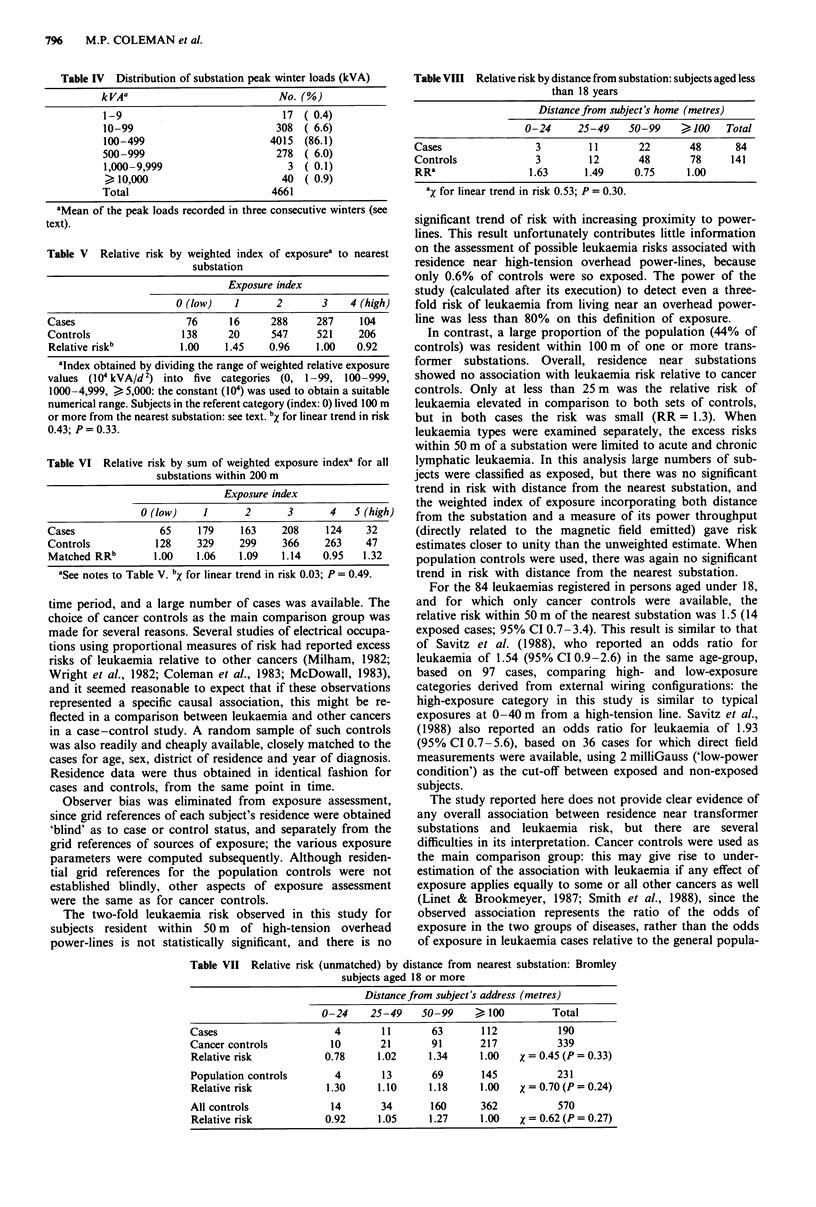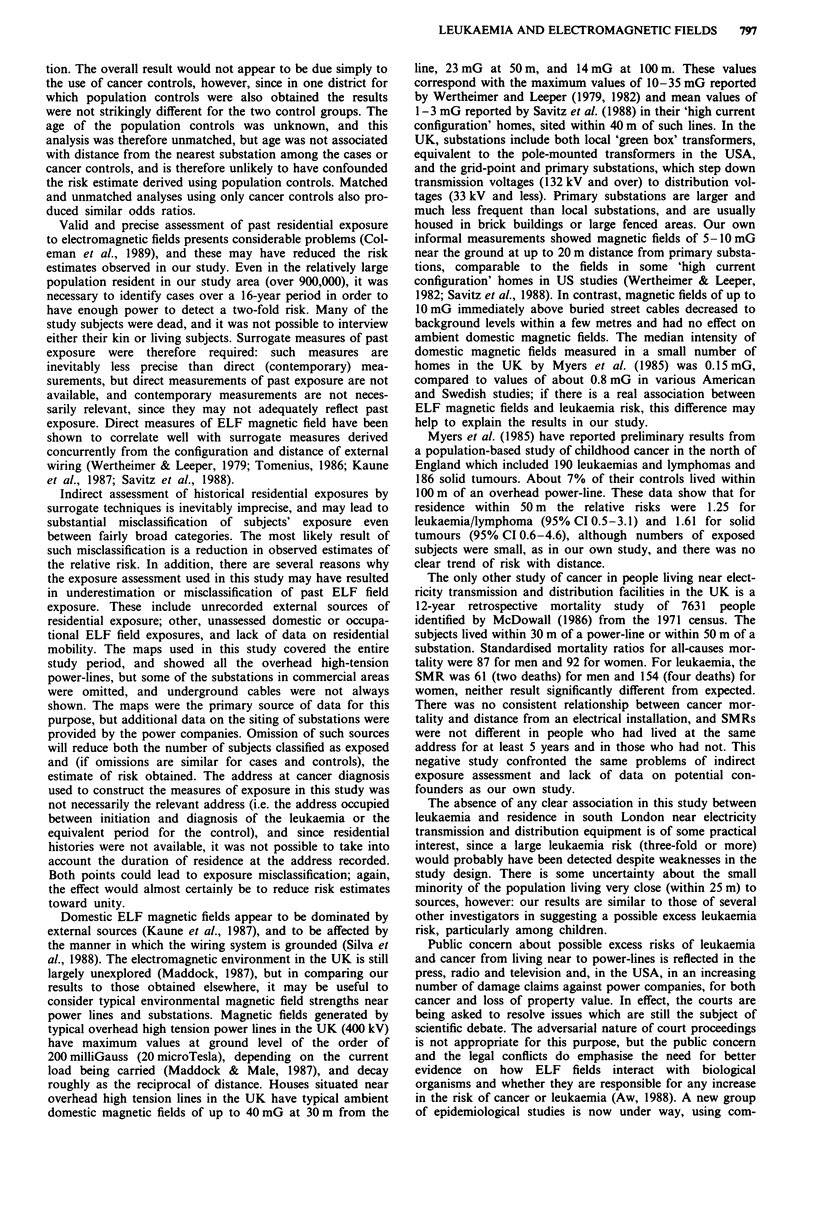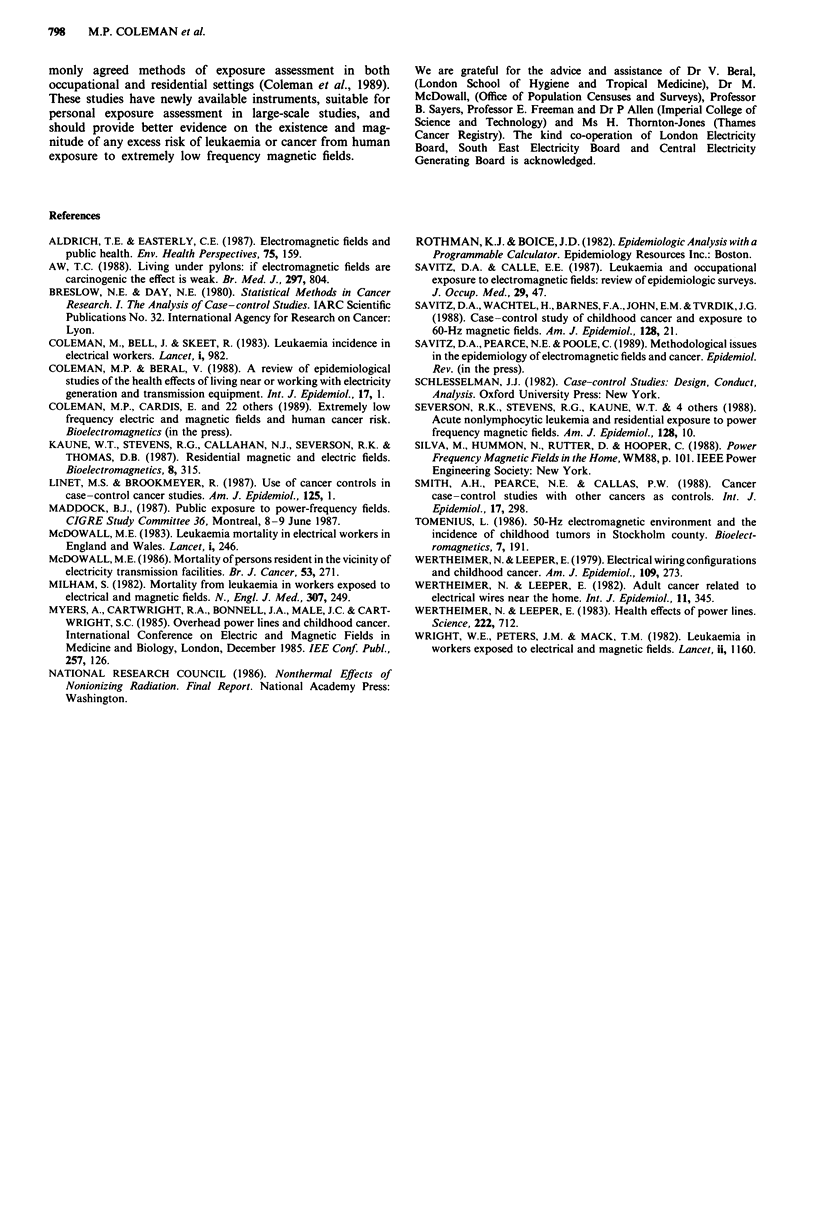# Leukaemia and residence near electricity transmission equipment: a case-control study.

**DOI:** 10.1038/bjc.1989.362

**Published:** 1989-11

**Authors:** M. P. Coleman, C. M. Bell, H. L. Taylor, M. Primic-Zakelj

**Affiliations:** International Agency for Research on Cancer, Lyon, France.

## Abstract

A population-based case-control study of leukaemia and residential proximity to electricity supply equipment has been carried out in south-east England. A total of 771 leukaemias was studied, matched for age, sex, year of diagnosis and district of residence to 1,432 controls registered with a solid tumour excluding lymphoma; 231 general population controls aged 18 and over from one part of the study area were also used. The potential for residential exposure to power frequency magnetic fields from power-lines and transformer substations was assessed indirectly from the distance, type and loading of the equipment near each subject's residence. Only 0.6% of subjects lived within 100 m of an overhead power-line, and the risk of leukaemia relative to cancer controls for residence within 100 m was 1.45 (95% confidence interval (CI) 0.54-3.88); within 50 m the relative risk was 2.0 but with a wider confidence interval (95% CI 0.4-9.0). Over 40% of subjects lived within 100 m of a substation, for which the relative risk of leukaemia was 0.99. Residence within 25 m carried a risk of 1.3 (95% CI 0.8-2.0). Weighted exposure indices incorporating measures of the current load carried by the substations did not materially alter these risks estimates. For persons aged less than 18 the relative risk of leukaemia from residence within 50 m of a substation was higher than in adults (PR = 1.5, 95% CI 0.7-3.4).


*Br. J. Cancer* (1989), **60**, 793–798 © The Macmillan Press Ltd., 1989

### Leukaemia and residence near electricity transmission equipment: a case–control study

M.P. Coleman[1], C.M.J. Bell[2], H.-L. Taylor[2] & M. Primic-Zakelj[3]

[1]*International Agency for Research on Cancer, 150 cours Albert-Thomas, 69372 Lyon Cedex 08, France;* [2]*London School of Hygiene & Tropical Medicine, Keppel Street, London, WC1E 7HT, UK; and* [3]*Cancer Registry of Slovenia, Institute of Oncology, Zaloska 2, 61000 Ljubljana, Yugoslavia.*

**Summary** A population-based case–control study of leukaemia and residential proximity to electricity supply equipment has been carried out in south-east England. A total of 771 leukaemias was studied, matched for age, sex, year of diagnosis and district of residence to 1,432 controls registered with a solid tumour excluding lymphoma; 231 general population controls aged 18 and over from one part of the study area were also used. The potential for residential exposure to power frequency magnetic fields from power-lines and transformer substations was assessed indirectly from the distance, type and loading of the equipment near each subject's residence. Only 0.6% of subjects lived within 100 m of an overhead power-line, and the risk of leukaemia relative to cancer controls for residence within 100 m was 1.45 (95% confidence interval (CI) 0.54–3.88); within 50 m the relative risk was 2.0 but with a wider confidence interval (95% CI 0.4–9.0). Over 40% of subjects lived within 100 m of a substation, for which the relative risk of leukaemia was 0.99. Residence within 25 m carried a risk of 1.3 (95% CI 0.8–2.0). Weighted exposure indices incorporating measures of the current load carried by the substations did not materially alter these risks estimates. For persons aged less than 18 the relative risk of leukaemia from residence within 50 m of a substation was higher than in adults (RR = 1.5, 95% CI 0.7–3.4).

Epidemiological evidence suggests a possible leukaemogenic effect in man from exposure to electromagnetic fields in the extremely low frequency range (ELF, 0–300 Hz), which includes the usual public electricity power supply frequencies (50–60 Hz). Three case–control studies have shown a two- to three-fold increase in leukaemia risk in persons who lived close to electricity power-lines and supply equipment (Wertheimer & Leeper, 1979, 1982; Savitz *et al.*, 1988). Two studies showed no association (Tomenius, 1986; Severson *et al.*, 1988), although the study by Tomenius showed a two-fold risk of all cancers. The subjects' exposure to ELF fields was categorised indirectly in these studies by the type and proximity of electricity transmission and distribution equipment variously within 40–150 m of the subject's home. In addition, ELF magnetic field intensities were measured directly at all addresses in one study (Tomenius, 1986), and at most addresses in the two recent studies (Severson *et al.*, 1988; Savitz *et al.*, 1988).

A number of studies of men likely to be exposed occupationally to power frequency electromagnetic fields have also suggested a raised risk of leukaemia, especially acute myeloid leukaemia (see Aldrich & Easterly, 1987; Savitz & Calle, 1987; Coleman & Beral, 1988). Interpretation of the evidence is made difficult by the complexity and ubiquity of human exposure to man-made ELF fields in modern society, and by the difficulty of obtaining satisfactory retrospective measures of this exposure. The National Research Council (NRC, 1986) and the reviews cited have emphasised the need for further human cancer studies, particularly of leukaemia, in relation to ELF magnetic field exposure.

We have conducted a population-based case–control study in south-east England to test the hypothesis that residential proximity to electricity transmission and distribution equipment may increase the risk of leukaemia. The purpose of the study was to address the practical question of whether typical public exposures related to the UK power supply system were associated with an excess leukaemia risk. In contrast to Sweden and the USA, from where studies have been reported so far, urban electricity distribution in the UK is almost entirely by underground cable. Only high-tension transmission lines in rural areas, operated at 132 kV or more, are placed above ground on pylons, as elsewhere. The two types of electricity supply equipment considered in this study were thus overhead powerlines rated at 132 kV and above, which constitute the main transmission network above ground, and transformer substations, which reduce the voltage in various steps to the local supply voltage (usually 240 V).

### Materials and methods

In order to obtain a sufficiently large and unbiased sample of leukaemia cases, the records of an established population-based cancer registry for a densely populated area were used. Cases were all persons registered with incident leukaemia by the Thames Cancer Registry during the period 1965–80 and resident in one of four adjacent London boroughs (Bromley, Croydon, Merton and Sutton; see Figure 1) which comprised the study area. Over 99% of leukaemias registered are histologically confirmed. The study area contains both urban and semi-rural sectors. There were no boundary changes during the study period, and the 1981 census population was 931,000. Most of the dwellings are houses of 1–3 floors or apartment buildings of 2–5 floors; high-rise blocks of 10–12 floors are infrequent.

Two groups of controls were used. The first group ('cancer controls') was identified from the same registry as the cases. Two controls were randomly selected among all persons registered with a solid tumour (excluding lymphoma) who could be individually matched to each case for sex, exact age in years and year of diagnosis. Controls were also required to be living in the same borough of residence as the case, as a partial surrogate for urban–rural and socio-economic status. Where possible, a reserve control was also selected for each case.

The second control group ('population controls') comprised a random sample of the general population aged 18 and over, drawn from the electoral roll for Bromley for 1975. Electoral registration is not compulsory, but largely complete. The roll does not state age or sex, and the population control series was therefore compared to Bromley cases aged 18 or over in an unmatched analysis. The same subset of the cancer controls (Bromley, aged 18 or over) was also analysed in this way, in order to provide a direct contrast between results obtained with the two groups of controls.

Correspondence: M.P. Coleman.
Received 2 March 1989; and in revised form 31 July 1989.

*Exposure assessment*

The electricity transmission and distribution network in the study area has changed little since 1962. Overhead high-tension power-lines at 132, 275 or 400 kV provide the main visible transmission network in the study area; some of the high voltage distribution is by underground cable. Voltage reduction transformers (33 to 11 kV, and 11 kV to supply voltage) were the most common type of electricity supply equipment, occurring every few hundred metres, more densely in built-up areas. These ground-level distribution transformers are roughly equivalent to the pole-mounted transformers in the USA.

It was not possible to obtain direct measurements of field intensity for this study, or of duration of exposure, since interviews and residence histories would have been required, and it was a condition of access to Thames Cancer Registry data for the study that no contact would be made with study subjects or their kin. Further, many subjects had been diagnosed up to 18 years before data collection began, and many were known to be dead. Instead we assessed the potential for past residential exposure of cases and controls to power frequency magnetic fields indirectly, from the distance, type and power loading of each component of the electricity supply equipment (source) within 100 m of each subject's home. Several exposure measures were then derived, and subjects were grouped into four or five ranked categories of each measure for analysis. Such measures are similar in principle to the 'wire configuration codes' first used by Wertheimer and Leeper (1979, 1982) and later by most other workers. These indirect measures have been shown to correlate well with concurrent direct measurements of ELF fields inside the home (Kaune *et al.*, 1987), and several authors have suggested that wire configuration codes may be a better surrogate for historical exposure to ELF fields emitted by power-lines than direct measurement at a single recent point in time (Savitz *et al.*, 1988, 1989; Wertheimer & Leeper, 1983). Savitz *et al.* (1988) found that for leukaemia there was a stronger association with wire codes than with direct contemporary measures of field intensity. Severson *et al.* (1988), however, found no association between either wire codes or direct measures of field intensity and the risk of acute non-lymphocytic leukaemia in adults.

The intensity of magnetic fields emitted by electrical equipment increases with the electric current flowing and decreases with the distance from the equipment. For linear sources such as overhead power-lines, the field intensity is inversely proportional to the distance from the line. The magnitude and spatial distribution of the field emitted by transformers and other equipment depends on the precise configuration of the electrical conductors in the equipment and the complex paths of current in the vicinity, however, and cannot be readily calculated, but tends to fall more rapidly than the reciprocal of distance. The conductors used in underground cables in the UK, rated at up to 132 kV, are intertwined in a helical arrangement which results in a very small net unbalanced current, and the small fields which they produce decay rapidly with distance (roughly as the inverse cube). They were excluded from exposure assessment.

The distance between each subject's residence and each source within 100 m was computed from their respective geographical grid references. Ten-metre grid references were recorded for the address of each subject at diagnosis (or on the electoral roll) from large-scale contemporary Ordnance Survey maps of the study area, which show individually numbered houses and street names. The 'centre of gravity' of the building was used as a reference point. The maps were of two scales: 1 in 1,250 (8 mm = 10 m) and 1 in 2,500 (4 mm = 10 m). The $X$ and $Y$ co-ordinates were recorded with specially prepared scale devices enabling accuracy to within 5 m or less in each axis. Even though exposure variables were not obtained directly from the maps, the case or control status of the subject was concealed from the person recording the grid references, in order to avoid any possible bias in exposure assessment. It was not possible to establish residential grid references 'blindly' for the population controls.

The grid reference of every substation was recorded systematically from each of the 600 or more maps of the study area; for overhead power-lines the co-ordinates of every pylon along the path of the line were recorded. The distance from each subject's home to each substation within 100 m and to the span of any overhead line within 100 m was then computed from the grid references. Additional data on representative power loads carried by each substation were provided by the electricity supply authorities, permitting a weighted index of exposure to be computed. The weighting factor used, $w$, was the peak winter load in kilovolt-amps (kVA) recorded for each substation, averaged over three consecutive winters. This 'peak winter load' was the only available measure of the electrical power loads carried in the past by each substation, and while it does not enable any direct estimation of magnetic fields, it does provide a simple measure of the likely relative magnitude of field produced by substations within the study area.

The main index of exposure used in the analysis was the distance, $d$, of the subject's residence from the nearest source, categorised as 0–24, 25–49, 50–99 and $\geqslant 100$ m, the last being the referent category. Overhead lines and substations were analysed separately. Other exposure indices were examined, including inverse measures of distance ($1/d$ for overhead lines and $1/d^2$ for substations), both for the nearest source (within 100 m) and for all sources within 200 m. Weighted indices ($w/d$ and $w/d^2$) were also used for the nearest source and for all sources within 200 m.

Matched analyses were done by conditional logistic regression for case–control studies with a variable matching ratio and categorical exposure variables (Breslow & Day, 1980). Unmatched analyses were done with the Mantel extension procedure, and test-based confidence intervals, using programs provided by Rothman and Boice (1982).

The limited available data on residential proximity to electricity supply equipment suggested that about 1% of urban populations in the UK might live within 100 m of a source (M.E. McDowall, personal communication). To estimate the likely power of the study in advance, residence within 100 m of a source was arbitrarily defined as a dichotomous 'exposure', and power calculations were based on two controls per case, a one-sided 5% significance level, and the expected availability of at least 650 cases for assessment. These calculations suggested that the study would have 90% power to detect a two-fold risk if 3% of the population were 'exposed', but only 80% power to detect a 2.5-fold risk if as few as 1% of the population were 'exposed' (Schlesselmann, 1982).

### Results

We identified 811 eligible cases of leukaemia registered in the study area in the period 1965–80, and 1,614 cancer controls. Thirty-six cases were excluded, each with both controls, because the address recorded at registration of the case could not be located; for 106 primary controls similarly excluded there was no eligible reserve. Four other cases were excluded because none of their controls could be located for use in matched analyses, and four controls were excluded on their second occurrence in the control group with a different primary tumour. Thus, 771 cases (95% of those eligible) were available for analysis, 110 matched to one control and 661 matched to two controls, a total of 1,432 controls (89% of those eligible). Only three (0.4%) of the 771 leukaemias were histologically unclassified. The distribution of leukaemia types by district of residence is given in Table I. The population control group comprised 254 persons from the 1975 Bromley electoral roll, of whom the addresses of 231 (91%) were located and assessed for exposure.

The odds ratios for leukaemia by distance from the nearest source are shown in Table II. High-tension overhead power-lines (132 or 275 kV) cross only a few residential zones in the

**Table I** Distribution of subjects by leukaemia type and borough

| | Borough | | | | |
|---|---|---|---|---|---|
| *Leukaemia type* | *Bromley* | *Croydon* | *Merton* | *Sutton* | *Total (%)* |
| Acute lymphoid | 32 | 42 | 20 | 22 | 116 (15) |
| Chronic lymphoid | 66 | 107 | 55 | 57 | 285 (37) |
| Acute myeloid | 81 | 85 | 38 | 44 | 248 (32) |
| Chronic myeloid | 30 | 54 | 20 | 15 | 119 (15) |
| Unclassified | | | | | 3 |
| All cases | 209 | 288 | 133 | 138 | 771 |
| Cancer controls | 368 | 546 | 284 | 234 | 1432 |
| Population controls | 231 | – | – | – | 231 |

**Table II** Relative risk by distance from source: cancer controls

| | *Distance from subject's address to nearest source (metres)* | | | | |
|---|---|---|---|---|---|
| | *0–24* | *25–49* | *50–99* | *≥100* | *Total* |
| *Power lines* | | | | | |
| Cases | 1 | 2 | 4 | 764 | 771 |
| Controls | 1 | 2 | 6 | 1423 | 1432 |
| Matched RR | 2.00 | 2.00 | 1.33 | 1.00 | |
| *Substations* | | | | | |
| Cases | 35 | 62 | 244 | 430 | 771 |
| Controls | 51 | 129 | 456 | 796 | 1432 |
| Matched RR | 1.26 | 0.89 | 0.99 | 1.00 | |

study area (Figure 1), and only nine (0.6%) of the controls lived within 100 m of such a power-line at cancer registration. The relative risk of leukaemia for residence within 100 m was 1.45 (95% CI 0.54–3.88). This excess is not statistically significant, and depends on only seven exposed cases. Residence within 50 m of a power-line was associated with a risk of 2.0 (95% CI 0.4–9.0), but this risk depends on only three exposed cases, and the trend of increasing risk with proximity is not significant ($P = 0.20$). Alternative exposure measures, including a weighted measure incorporating the line voltage rating, made no material difference to the risk estimates. In view of the rarity of residential exposure to overhead power-lines in this population, no more detailed analysis was feasible.

More than 4,600 transformer substations were identified in the study area, and 44% of the cancer controls lived within 100 m of at least one substation at cancer registration. Residence within 100 m of a substation was not associated with an excess leukaemia risk (RR = 0.99). Analysis by distance from the nearest substation revealed no clear pattern of risk (Table II), although the closest distance category also had the highest risk (RR = 1.3, 95% CI 0.81–1.98). There was a slight increase in the risk of acute lymphatic leukaemia within 50 m of the nearest substation (Table III). There was no consistent pattern of risk between the leukaemia types, and in particular there was no suggestion of an increased risk of acute myeloid leukaemia.

The peak winter load of each substation (in kVA) was used to provide a weighted exposure variable. The great

**Figure 1** Map of study area in south London, showing main overhead high tension power lines (OHL).

majority (86%) of substations were of similar type (11 kV reduced to supply voltage) and had similar recorded peak winter loads (Table IV); unknown kVA values for 21 (0.5%) substations were set to the mean load (335 kVA) in the analysis. An example analysis using such a weighted index of exposure (load/$d^2$ for the substation nearest to the home) is shown in Table V. There was no evidence of an excess leukaemia risk. The same weighted index was then added for all substations within 200 m of each subject's home as a cumulative measure (Table VI). The category with the highest exposure index had the largest risk, but this was still small (RR = 1.3, 95% CI 0.8–2.3)

Results obtained using population controls, for Bromley only, are shown in Table VII. These controls were compared with the 190 cases (91%) and 339 cancer controls (92%) resident in Bromley who were aged 18 or more. Similar proportions of both control groups lived within 100 m of at least one substation. Risk estimates within 50 m of the nearest substation were higher with population controls (RR = 1.14, 95% CI 0.55–2.39) than with cancer controls (RR = 0.85, 95% CI 0.45–1.62), but the trend of leukaemia risk with proximity to the nearest substation was not significant with either control group. None of these subjects lived within 100 m of a power-line.

In an analysis covering the entire study area but restricted to subjects aged less than 18 years (Table VIII), there were 84 leukaemia cases (11% of total) and 141 cancer controls (10%). There is a suggestion that residence within 25 or 50 m of a substation is associated with a small increase in risk, but this trend is not statistically significant. Sixty-three (45%) of the controls lived within 100 m of a substation and the relative risk of leukaemia for this exposure was 0.93 (95% CI 0.54–1.60); for residence within 50 m the relative risk compared to the referent category was 1.52 (95% CI 0.67–3.42). Only one case and one control were resident within 100 m of an overhead power-line.

### Discussion

The design of this study provided several advantages over earlier studies in selection of the study subjects and avoidance of bias in exposure assessment, but exposure assessment was crude and indirect, and caution is required when interpreting the results.

The leukaemia cases are a virtually complete population sample of incident cases from a well-defined territory and

**Table III** Unmatched relative risk (no. of cases) by type of leukaemia and distance from nearest substation: cancer controls

| | *Distance from subject's address (metres)* | | | | | |
|---|---|---|---|---|---|---|
| *Type of leukaemia* | *0–24* | *25–49* | *50–99* | *≥100* | $\chi^b$ | *P* |
| Acute lymphatic | 1.76 ( 7) | 1.39 (14) | 0.93 ( 33) | 1.00 ( 62) | 1.31 | 0.10 |
| Chronic lymphatic | 1.61 (16) | 0.96 (24) | 1.01 ( 90) | 1.00 (155) | 0.90 | 0.18 |
| Acute myeloid | 0.98 ( 9) | 0.73 (17) | 0.95 ( 78) | 1.00 (144) | –0.82 | >0.50 |
| Chronic myeloid | 0.70 ( 3) | 0.65 ( 7) | 1.09 ( 42) | 1.00 ( 67) | 0.79 | 0.22 |
| All types[a] | 1.28 (35) | 0.89 (62) | 0.99 (243) | 1.00 (428) | 0.25 | 0.40 |
| No. of controls | 51 | 129 | 456 | 796 | | |

[a]All specified types: small differences in risk from Table II are due to exclusion of the three unclassified leukaemias, two of which are in the referent exposure category. [b]Test for linear trend in risk.

**Table IV** Distribution of substation peak winter loads (kVA)

| *kVA*[a] | *No. (%)* |
|---|---|
| 1–9 | 17 ( 0.4) |
| 10–99 | 308 ( 6.6) |
| 100–499 | 4015 (86.1) |
| 500–999 | 278 ( 6.0) |
| 1,000–9,999 | 3 ( 0.1) |
| $\geqslant$10,000 | 40 ( 0.9) |
| Total | 4661 |

[a]Mean of the peak loads recorded in three consecutive winters (see text).

**Table V** Relative risk by weighted index of exposure[a] to nearest substation

| | *Exposure index* | | | | |
|---|---|---|---|---|---|
| | *0 (low)* | *1* | *2* | *3* | *4 (high)* |
| Cases | 76 | 16 | 288 | 287 | 104 |
| Controls | 138 | 20 | 547 | 521 | 206 |
| Relative risk[b] | 1.00 | 1.45 | 0.96 | 1.00 | 0.92 |

[a]Index obtained by dividing the range of weighted relative exposure values ($10^4$ kVA/$d^2$) into five categories (0, 1–99, 100–999, 1000–4,999, $\geqslant$5,000: the constant ($10^4$) was used to obtain a suitable numerical range. Subjects in the referent category (index: 0) lived 100 m or more from the nearest substation: see text. [b]$\chi$ for linear trend in risk 0.43; $P = 0.33$.

**Table VI** Relative risk by sum of weighted exposure index[a] for all substations within 200 m

| | *Exposure index* | | | | | |
|---|---|---|---|---|---|---|
| | *0 (low)* | *1* | *2* | *3* | *4* | *5 (high)* |
| Cases | 65 | 179 | 163 | 208 | 124 | 32 |
| Controls | 128 | 329 | 299 | 366 | 263 | 47 |
| Matched RR[b] | 1.00 | 1.06 | 1.09 | 1.14 | 0.95 | 1.32 |

[a]See notes to Table V. [b]$\chi$ for linear trend in risk 0.03; $P = 0.49$.

time period, and a large number of cases was available. The choice of cancer controls as the main comparison group was made for several reasons. Several studies of electrical occupations using proportional measures of risk had reported excess risks of leukaemia relative to other cancers (Milham, 1982; Wright *et al.*, 1982; Coleman *et al.*, 1983; McDowall, 1983), and it seemed reasonable to expect that if these observations represented a specific causal association, this might be reflected in a comparison between leukaemia and other cancers in a case–control study. A random sample of such controls was also readily and cheaply available, closely matched to the cases for age, sex, district of residence and year of diagnosis. Residence data were thus obtained in identical fashion for cases and controls, from the same point in time.

Observer bias was eliminated from exposure assessment, since grid references of each subject's residence were obtained 'blind' as to case or control status, and separately from the grid references of sources of exposure; the various exposure parameters were computed subsequently. Although residential grid references for the population controls were not established blindly, other aspects of exposure assessment were the same as for cancer controls.

The two-fold leukaemia risk observed in this study for subjects resident within 50 m of high-tension overhead power-lines is not statistically significant, and there is no significant trend of risk with increasing proximity to power-lines. This result unfortunately contributes little information on the assessment of possible leukaemia risks associated with residence near high-tension overhead power-lines, because only 0.6% of controls were so exposed. The power of the study (calculated after its execution) to detect even a three-fold risk of leukaemia from living near an overhead power-line was less than 80% on this definition of exposure.

In contrast, a large proportion of the population (44% of controls) was resident within 100 m of one or more transformer substations. Overall, residence near substations showed no association with leukaemia risk relative to cancer controls. Only at less than 25 m was the relative risk of leukaemia elevated in comparison to both sets of controls, but in both cases the risk was small (RR = 1.3). When leukaemia types were examined separately, the excess risks within 50 m of a substation were limited to acute and chronic lymphatic leukaemia. In this analysis large numbers of subjects were classified as exposed, but there was no significant trend in risk with distance from the nearest substation, and the weighted index of exposure incorporating both distance from the substation and a measure of its power throughput (directly related to the magnetic field emitted) gave risk estimates closer to unity than the unweighted estimate. When population controls were used, there was again no significant trend in risk with distance from the nearest substation.

For the 84 leukaemias registered in persons aged under 18, and for which only cancer controls were available, the relative risk within 50 m of the nearest substation was 1.5 (14 exposed cases; 95% CI 0.7–3.4). This result is similar to that of Savitz *et al.* (1988), who reported an odds ratio for leukaemia of 1.54 (95% CI 0.9–2.6) in the same age-group, based on 97 cases, comparing high- and low-exposure categories derived from external wiring configurations: the high-exposure category in this study is similar to typical exposures at 0–40 m from a high-tension line. Savitz *et al.*, (1988) also reported an odds ratio for leukaemia of 1.93 (95% CI 0.7–5.6), based on 36 cases for which direct field measurements were available, using 2 milliGauss ('low-power condition') as the cut-off between exposed and non-exposed subjects.

The study reported here does not provide clear evidence of any overall association between residence near transformer substations and leukaemia risk, but there are several difficulties in its interpretation. Cancer controls were used as the main comparison group: this may give rise to under-estimation of the association with leukaemia if any effect of exposure applies equally to some or all other cancers as well (Linet & Brookmeyer, 1987; Smith *et al.*, 1988), since the observed association represents the ratio of the odds of exposure in the two groups of diseases, rather than the odds of exposure in leukaemia cases relative to the general popula-

**Table VIII** Relative risk by distance from substation: subjects aged less than 18 years

| | *Distance from subject's home (metres)* | | | | |
|---|---|---|---|---|---|
| | *0–24* | *25–49* | *50–99* | *$\geqslant$100* | *Total* |
| Cases | 3 | 11 | 22 | 48 | 84 |
| Controls | 3 | 12 | 48 | 78 | 141 |
| RR[a] | 1.63 | 1.49 | 0.75 | 1.00 | |

[a]$\chi$ for linear trend in risk 0.53; $P = 0.30$.

**Table VII** Relative risk (unmatched) by distance from nearest substation: Bromley subjects aged 18 or more

| | *Distance from subject's address (metres)* | | | | |
|---|---|---|---|---|---|
| | *0–24* | *25–49* | *50–99* | $\geqslant$100 | Total |
| Cases | 4 | 11 | 63 | 112 | 190 |
| Cancer controls | 10 | 21 | 91 | 217 | 339 |
| Relative risk | 0.78 | 1.02 | 1.34 | 1.00 | $\chi = 0.45$ ($P = 0.33$) |
| Population controls | 4 | 13 | 69 | 145 | 231 |
| Relative risk | 1.30 | 1.10 | 1.18 | 1.00 | $\chi = 0.70$ ($P = 0.24$) |
| All controls | 14 | 34 | 160 | 362 | 570 |
| Relative risk | 0.92 | 1.05 | 1.27 | 1.00 | $\chi = 0.62$ ($P = 0.27$) |

tion. The overall result would not appear to be due simply to the use of cancer controls, however, since in one district for which population controls were also obtained the results were not strikingly different for the two control groups. The age of the population controls was unknown, and this analysis was therefore unmatched, but age was not associated with distance from the nearest substation among the cases or cancer controls, and is therefore unlikely to have confounded the risk estimate derived using population controls. Matched and unmatched analyses using only cancer controls also produced similar odds ratios.

Valid and precise assessment of past residential exposure to electromagnetic fields presents considerable problems (Coleman *et al.*, 1989), and these may have reduced the risk estimates observed in our study. Even in the relatively large population resident in our study area (over 900,000), it was necessary to identify cases over a 16-year period in order to have enough power to detect a two-fold risk. Many of the study subjects were dead, and it was not possible to interview either their kin or living subjects. Surrogate measures of past exposure were therefore required: such measures are inevitably less precise than direct (contemporary) measurements, but direct measurements of past exposure are not available, and contemporary measurements are not necessarily relevant, since they may not adequately reflect past exposure. Direct measures of ELF magnetic field have been shown to correlate well with surrogate measures derived concurrently from the configuration and distance of external wiring (Wertheimer & Leeper, 1979; Tomenius, 1986; Kaune *et al.*, 1987; Savitz *et al.*, 1988).

Indirect assessment of historical residential exposures by surrogate techniques is inevitably imprecise, and may lead to substantial misclassification of subjects' exposure even between fairly broad categories. The most likely result of such misclassification is a reduction in observed estimates of the relative risk. In addition, there are several reasons why the exposure assessment used in this study may have resulted in underestimation or misclassification of past ELF field exposure. These include unrecorded external sources of residential exposure; other, unassessed domestic or occupational ELF field exposures, and lack of data on residential mobility. The maps used in this study covered the entire study period, and showed all the overhead high-tension power-lines, but some of the substations in commercial areas were omitted, and underground cables were not always shown. The maps were the primary source of data for this purpose, but additional data on the siting of substations were provided by the power companies. Omission of such sources will reduce both the number of subjects classified as exposed and (if omissions are similar for cases and controls), the estimate of risk obtained. The address at cancer diagnosis used to construct the measures of exposure in this study was not necessarily the relevant address (i.e. the address occupied between initiation and diagnosis of the leukaemia or the equivalent period for the control), and since residential histories were not available, it was not possible to take into account the duration of residence at the address recorded. Both points could lead to exposure misclassification; again, the effect would almost certainly be to reduce risk estimates toward unity.

Domestic ELF magnetic fields appear to be dominated by external sources (Kaune *et al.*, 1987), and to be affected by the manner in which the wiring system is grounded (Silva *et al.*, 1988). The electromagnetic environment in the UK is still largely unexplored (Maddock, 1987), but in comparing our results to those obtained elsewhere, it may be useful to consider typical environmental magnetic field strengths near power lines and substations. Magnetic fields generated by typical overhead high tension power lines in the UK (400 kV) have maximum values at ground level of the order of 200 milliGauss (20 microTesla), depending on the current load being carried (Maddock & Male, 1987), and decay roughly as the reciprocal of distance. Houses situated near overhead high tension lines in the UK have typical ambient domestic magnetic fields of up to 40 mG at 30 m from the line, 23 mG at 50 m, and 14 mG at 100 m. These values correspond with the maximum values of 10–35 mG reported by Wertheimer and Leeper (1979, 1982) and mean values of 1–3 mG reported by Savitz *et al.* (1988) in their 'high current configuration' homes, sited within 40 m of such lines. In the UK, substations include both local 'green box' transformers, equivalent to the pole-mounted transformers in the USA, and the grid-point and primary substations, which step down transmission voltages (132 kV and over) to distribution voltages (33 kV and less). Primary substations are larger and much less frequent than local substations, and are usually housed in brick buildings or large fenced areas. Our own informal measurements showed magnetic fields of 5–10 mG near the ground at up to 20 m distance from primary substations, comparable to the fields in some 'high current configuration' homes in US studies (Wertheimer & Leeper, 1982; Savitz *et al.*, 1988). In contrast, magnetic fields of up to 10 mG immediately above buried street cables decreased to background levels within a few metres and had no effect on ambient domestic magnetic fields. The median intensity of domestic magnetic fields measured in a small number of homes in the UK by Myers *et al.* (1985) was 0.15 mG, compared to values of about 0.8 mG in various American and Swedish studies; if there is a real association between ELF magnetic fields and leukaemia risk, this difference may help to explain the results in our study.

Myers *et al.* (1985) have reported preliminary results from a population-based study of childhood cancer in the north of England which included 190 leukaemias and lymphomas and 186 solid tumours. About 7% of their controls lived within 100 m of an overhead power-line. These data show that for residence within 50 m the relative risks were 1.25 for leukaemia/lymphoma (95% CI 0.5–3.1) and 1.61 for solid tumours (95% CI 0.6–4.6), although numbers of exposed subjects were small, as in our own study, and there was no clear trend of risk with distance.

The only other study of cancer in people living near electricity transmission and distribution facilities in the UK is a 12-year retrospective mortality study of 7631 people identified by McDowall (1986) from the 1971 census. The subjects lived within 30 m of a power-line or within 50 m of a substation. Standardised mortality ratios for all-causes mortality were 87 for men and 92 for women. For leukaemia, the SMR was 61 (two deaths) for men and 154 (four deaths) for women, neither result significantly different from expected. There was no consistent relationship between cancer mortality and distance from an electrical installation, and SMRs were not different in people who had lived at the same address for at least 5 years and in those who had not. This negative study confronted the same problems of indirect exposure assessment and lack of data on potential confounders as our own study.

The absence of any clear association in this study between leukaemia and residence in south London near electricity transmission and distribution equipment is of some practical interest, since a large leukaemia risk (three-fold or more) would probably have been detected despite weaknesses in the study design. There is some uncertainty about the small minority of the population living very close (within 25 m) to sources, however: our results are similar to those of several other investigators in suggesting a possible excess leukaemia risk, particularly among children.

Public concern about possible excess risks of leukaemia and cancer from living near to power-lines is reflected in the press, radio and television and, in the USA, in an increasing number of damage claims against power companies, for both cancer and loss of property value. In effect, the courts are being asked to resolve issues which are still the subject of scientific debate. The adversarial nature of court proceedings is not appropriate for this purpose, but the public concern and the legal conflicts do emphasise the need for better evidence on how ELF fields interact with biological organisms and whether they are responsible for any increase in the risk of cancer or leukaemia (Aw, 1988). A new group of epidemiological studies is now under way, using commonly agreed methods of exposure assessment in both occupational and residential settings (Coleman *et al.*, 1989). These studies have newly available instruments, suitable for personal exposure assessment in large-scale studies, and should provide better evidence on the existence and magnitude of any excess risk of leukaemia or cancer from human exposure to extremely low frequency magnetic fields.

We are grateful for the advice and assistance of Dr V. Beral, (London School of Hygiene and Tropical Medicine), Dr M. McDowall, (Office of Population Censuses and Surveys), Professor B. Sayers, Professor E. Freeman and Dr P Allen (Imperial College of Science and Technology) and Ms H. Thornton-Jones (Thames Cancer Registry). The kind co-operation of London Electricity Board, South East Electricity Board and Central Electricity Generating Board is acknowledged.

### References

ALDRICH, T.E. & EASTERLY, C.E. (1987). Electromagnetic fields and public health. *Env. Health Perspectives*, **75**, 159.

AW, T.C. (1988). Living under pylons: if electromagnetic fields are carcinogenic the effect is weak. *Br. Med. J.*, **297**, 804.

BRESLOW, N.E. & DAY, N.E. (1980). *Statistical Methods in Cancer Research. I. The Analysis of Case–control Studies*. IARC Scientific Publications No. 32. International Agency for Research on Cancer: Lyon.

COLEMAN, M., BELL, J. & SKEET, R. (1983). Leukaemia incidence in electrical workers. *Lancet*, **i**, 982.

COLEMAN, M.P. & BERAL, V. (1988). A review of epidemiological studies of the health effects of living near or working with electricity generation and transmission equipment. *Int. J. Epidemiol.*, **17**, 1.

COLEMAN, M.P., CARDIS, E. and 22 others (1989). Extremely low frequency electric and magnetic fields and human cancer risk. *Bioelectromagnetics* (in the press).

KAUNE, W.T., STEVENS, R.G., CALLAHAN, N.J., SEVERSON, R.K. & THOMAS, D.B. (1987). Residential magnetic and electric fields. *Bioelectromagnetics*, **8**, 315.

LINET, M.S. & BROOKMEYER, R. (1987). Use of cancer controls in case–control cancer studies. *Am. J. Epidemiol.*, **125**, 1.

MADDOCK, B.J., (1987). Public exposure to power-frequency fields. *CIGRE Study Committee 36*, Montreal, 8–9 June 1987.

McDOWALL, M.E. (1983). Leukaemia mortality in electrical workers in England and Wales. *Lancet*, **i**, 246.

McDOWALL, M.E. (1986). Mortality of persons resident in the vicinity of electricity transmission facilities. *Br. J. Cancer*, **53**, 271.

MILHAM, S. (1982). Mortality from leukaemia in workers exposed to electrical and magnetic fields. *N., Engl. J. Med.*, **307**, 249.

MYERS, A., CARTWRIGHT, R.A., BONNELL, J.A., MALE, J.C. & CARTWRIGHT, S.C. (1985). Overhead power lines and childhood cancer. International Conference on Electric and Magnetic Fields in Medicine and Biology, London, December 1985. *IEE Conf. Publ.*, **257**, 126.

NATIONAL RESEARCH COUNCIL (1986). *Nonthermal Effects of Nonionizing Radiation. Final Report*. National Academy Press: Washington.

ROTHMAN, K.J. & BOICE, J.D. (1982). *Epidemiologic Analysis with a Programmable Calculator*. Epidemiology Resources Inc.: Boston.

SAVITZ, D.A. & CALLE, E.E. (1987). Leukaemia and occupational exposure to electromagnetic fields: review of epidemiologic surveys. *J. Occup. Med.*, **29**, 47.

SAVITZ, D.A., WACHTEL, H., BARNES, F.A., JOHN, E.M. & TVRDIK, J.G. (1988). Case–control study of childhood cancer and exposure to 60-Hz magnetic fields. *Am. J. Epidemiol.*, **128**, 21.

SAVITZ, D.A., PEARCE, N.E. & POOLE, C. (1989). Methodological issues in the epidemiology of electromagnetic fields and cancer. *Epidemiol. Rev.* (in the press).

SCHLESSELMAN, J.J. (1982). *Case-control Studies: Design, Conduct, Analysis*. Oxford University Press: New York.

SEVERSON, R.K., STEVENS, R.G., KAUNE, W.T. & 4 others (1988). Acute nonlymphocytic leukemia and residential exposure to power frequency magnetic fields. *Am. J. Epidemiol.*, **128**, 10.

SILVA, M., HUMMON, N., RUTTER, D. & HOOPER, C. (1988). *Power Frequency Magnetic Fields in the Home*, WM88, p. 101. IEEE Power Engineering Society: New York.

SMITH, A.H., PEARCE, N.E. & CALLAS, P.W. (1988). Cancer case–control studies with other cancers as controls. *Int. J. Epidemiol.*, **17**, 298.

TOMENIUS, L. (1986). 50-Hz electromagnetic environment and the incidence of childhood tumors in Stockholm county. *Bioelectromagnetics*, **7**, 191.

WERTHEIMER, N. & LEEPER, E. (1979). Electrical wiring configurations and childhood cancer. *Am. J. Epidemiol.*, **109**, 273.

WERTHEIMER, N. & LEEPER, E. (1982). Adult cancer related to electrical wires near the home. *Int. J. Epidemiol.*, **11**, 345.

WERTHEIMER, N. & LEEPER, E. (1983). Health effects of power lines. *Science*, **222**, 712.

WRIGHT, W.E., PETERS, J.M. & MACK, T.M. (1982). Leukaemia in workers exposed to electrical and magnetic fields. *Lancet*, **ii**, 1160.